# Identification of Critical Residues of the Mycobacterial Dephosphocoenzyme A Kinase by Site-Directed Mutagenesis

**DOI:** 10.1371/journal.pone.0015228

**Published:** 2011-01-11

**Authors:** Guneet Walia, Komatireddy Gajendar, Avadhesha Surolia

**Affiliations:** 1 Molecular Biophysics Unit, Indian Institute of Science, Bangalore, India; 2 National Institute of Immunology, New Delhi, India; University of Hyderabad, India

## Abstract

Dephosphocoenzyme A kinase performs the transfer of the γ-phosphate of ATP to dephosphocoenzyme A, catalyzing the last step of coenzyme A biosynthesis. This enzyme belongs to the P-loop-containing NTP hydrolase superfamily, all members of which posses a three domain topology consisting of a CoA domain that binds the acceptor substrate, the nucleotide binding domain and the lid domain. Differences in the enzymatic organization and regulation between the human and mycobacterial counterparts, have pointed out the tubercular CoaE as a high confidence drug target (HAMAP database). Unfortunately the absence of a three-dimensional crystal structure of the enzyme, either alone or complexed with either of its substrates/regulators, leaves both the reaction mechanism unidentified and the chief players involved in substrate binding, stabilization and catalysis unknown. Based on homology modeling and sequence analysis, we chose residues in the three functional domains of the enzyme to assess their contributions to ligand binding and catalysis using site-directed mutagenesis. Systematically mutating the residues from the P-loop and the nucleotide-binding site identified Lys14 and Arg140 in ATP binding and the stabilization of the phosphoryl intermediate during the phosphotransfer reaction. Mutagenesis of Asp32 and Arg140 showed catalytic efficiencies less than 5–10% of the wild type, indicating the pivotal roles played by these residues in catalysis. Non-conservative substitution of the Leu114 residue identifies this leucine as the critical residue from the hydrophobic cleft involved in leading substrate, DCoA binding. We show that the mycobacterial enzyme requires the Mg^2+^ for its catalytic activity. The binding energetics of the interactions of the mutant enzymes with the substrates were characterized in terms of their enthalpic and entropic contributions by ITC, providing a complete picture of the effects of the mutations on activity. The properties of mutants defective in substrate recognition were consistent with the ordered sequential mechanism of substrate addition for CoaE.

## Introduction

Coenzyme A is an essential metabolic cofactor which participates in more than 9% of all enzymatic reactions in the cellular milieu (1). Therefore its biosynthetic pathway from species across the living spectrum has been the focus of considerable study. Coenzyme A is synthesized from its cellular precursors, pantothenate, cysteine and ATP in a series of five steps, initiated by pantothenate kinase (CoaA) which phosphorylates pantothenate to generate 4′-phosphopantothenate. The latter is then fused with a cysteine moiety by CoaB (4′phosphopantothenoylcsyteine synthetase) to generate 4′-phosphopantothenoylcysteine which is then decarboxylated by CoaC (4′-phosphopantothenoylcysteine decarboxylase). 4′-phosphopanthetheine, the product of the CoaC reaction is adenylated by 4′-phosphoadenylyltransferase (CoaD) to generate 4′-dephosphocoenzyme A. Dephosphocoenzyme A kinase (CoaE), the focus of the present study, utilizes ATP to phosphorylate dephosphocoenzyme A at the 3′-hydroxyl of the ribose moiety generating Coenzyme A.

CoaEs belong to the family of nucleotide and nucleoside kinases, which are members of the structural superfamily of NTP (nucleoside triphosphate) hydrolases according to the SCOP hierarchy (2). These enzymes share several common structural motifs despite having a negligible sequence similarity amongst each other. The chief amongst these is the P (phosphate-binding)-loop and the overall three-dimensional fold comprising of three domains. The latter together form the nucleotide-binding fold of these P-loop containing proteins; the five-stranded parallel β-sheet which forms the nucleotide-binding domain flanked on both sides by the α-helical substrate binding domain and the α-helical Lid domain. Another similarity among these enzymes is the demonstration of large domain movements during catalysis. Information on CoaEs from various sources has started emerging only very recently with crystal structures of dephosphocoenzyme A kinases being available from three bacterial sources, *Thermus thermophilus*, *E. coli* and *Haemophilus influenzae* (3–5). The *E. coli* enzyme is a crystallographic trimer while the *Thermus* and *Haemophilus* enzymes were monomeric in the crystal state.

The tubercular dephosphocoenzyme A kinase, encoded by the *coaE* gene, is a noticeable exception to the established paradigm of CoaEs as it possesses a unique domain at its C-terminus which plays a significant role in the folding of the full length enzyme (6). As reported in our previous study, the mycobacterial CoaE is also unique in that its activity is regulated by the metabolic effector, CTP, a property, as yet, unidentified for any CoaE from any species. The mycobacterial enzyme is vastly different from its human counterpart, the latter being a bifunctional enzyme that carries both the penultimate and last enzyme activities in a single polypeptide. Also, differences in regulation of the human and the pathogen enzymes point out the tubercular enzyme as a good antimycobacterial drug target (7). The absence of X-ray crystallographic data on the mycobacterial CoaE warrants a detailed analysis of the enzymatic architecture and mechanistic features employing other means. Owing to the low but significant levels of primary sequence identity as well as similarity in the docked model of the tubercular enzyme and the crystal structures of the *E. coli* enzyme, we tentatively assigned residues that may play crucial roles in substrate binding and catalysis. Therefore we chose to mutate residues implicated in critical roles in all the three domains of the mycobacterial CoaE, the CoA domain (Asp32, Leu114), the nucleotide binding domain (Lys14, Gly8, Thr7 and Arg140) and the lid domain (Arg140). These studies highlight the roles of specific amino acid residues in substrate binding and catalysis by replacement of the latter with residues that are functionally or structurally homologous and comparison with nonhomologous substitutions.

Therefore, in this study, residues indispensable for catalytic activity were identified. Mutants with reduced kinetic capabilities were analyzed further to study the effect of the amino acid alteration on the binding of the ligands. Based on the results presented, we have identified residues essential for leading substrate binding (Leu114), phosphate donor binding (Lys14), stabilization of the phosphoryl transfer reaction (Asp32 and Arg140) and catalysis (Asp32). Our studies have therefore helped to identify the previously unknown functional roles of highly conserved residues in dephosphocoenzyme A kinases. This study presents a thorough molecular dissection of the roles played by crucial amino acids of the protein and the results herein can serve as a good starting point for targeted drug development approaches.

## Results and Discussion

### Sequence Homology

**Figure 1:**
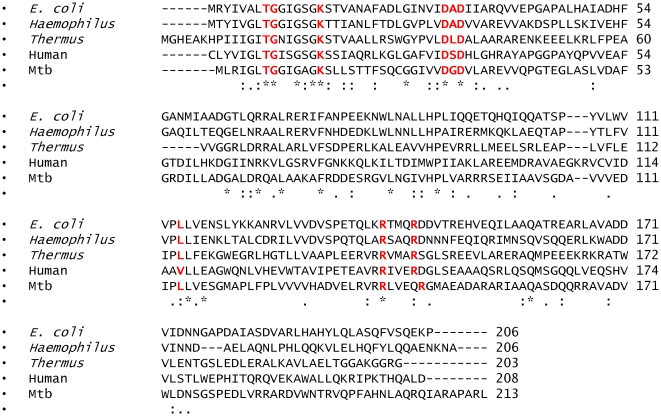
Multiple Sequence Alignment of the protein sequences of dephosphocoenzyme A kinases from *Escherichia coli* (UniProtKB ID P0A619), *Thermus thermophilus* (UniProtKB ID Q56416), *Haemophilus influenzae* (UniProtKB ID P44920), *Mycobacterium tuberculosis* (UniProtKB ID P63826) and the mammalian coenzyme A synthase (UniProtKB ID Q13057) carried out by the ClustalW algorithm. For purposes of clarity, only the CoaE domain of the mycobacterial and human enzymes have been shown. The residues chosen for mutagenesis have been highlighted and colored in red. Completely conserved residues are starred.

Figure 1 presents the primary sequence alignments of dephosphocoenzyme A kinases from different organisms in the regions of interest. A sequence alignment of all the proteins belonging to the NTP superfamily reveals considerable sequence conservation for all residues and motifs which are functionally important. The DXD motif has been highlighted as it is a highly conserved motif across all sequences with ∼80% identity for each pairwaise comparison in this region.

### Structural homology

**Figure 2:**
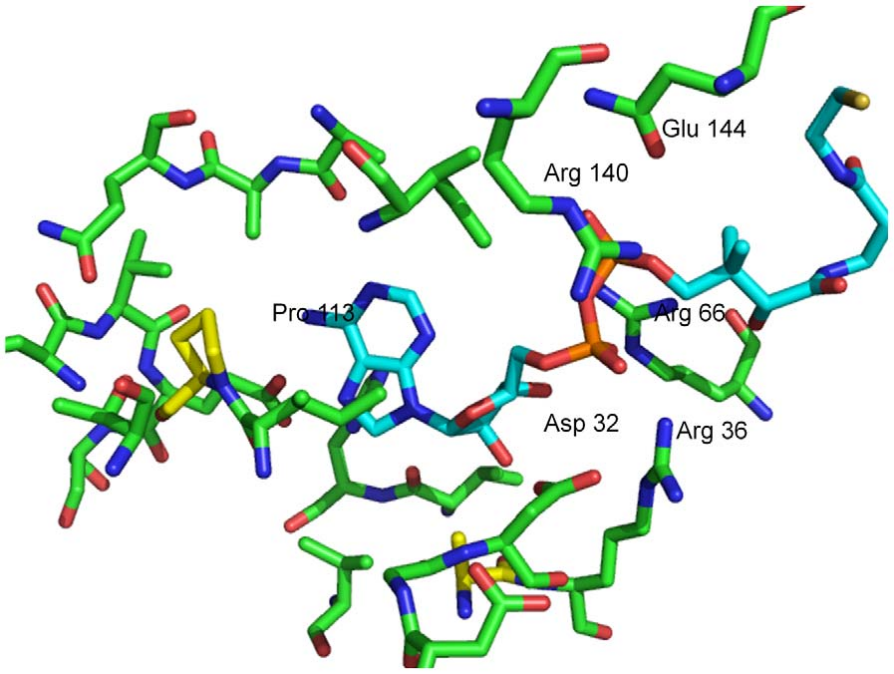
Homology modeled structure of the active site. The mycobacterial dephosphocoenzyme A kinase has been homology modeled and the leading substrate, DCoA has been docked at the active site. Protein residues are shown as green ball-and-stick models and the bound DCoA in cyan. The figure was generated in PyMol.

We have previously homology modeled the mycobacterial enzyme and its substrate, DCoA and cellular effector, CTP were docked on the enzyme (6). Figure 2 shows a view of DCoA docked on the active site of the modeled CoaE. The structural neighbors for the modeled CoaE in the three-dimensional similarity search using the DALI server (8) were the dephosphocoenzyme A kinase from *Thermus thermophilus* (PDB code 1UF9) (3), adenylate kinase from *Sulfolobus acidocaldarius* (PDB code 1NKS) (9), gluconate kinase from *E. coli* (PDB code 1KO5) (10), the uridylmonophosphate-cytidylmonophosphate kinase from *Dictyostelium discoideum* (PDB code 1UKE) (11) and thymidylate kinase from *Aquifex aeolicus* (PDB code 2PBR) (12). All these enzymes belong to the NTP hydrolases superfamily. Therefore conserved motifs from homologous structures and residues in the sequences were a good starting point for choosing amino acids for mutagenesis.

### CD and Fluorescence

**Figure 3:**
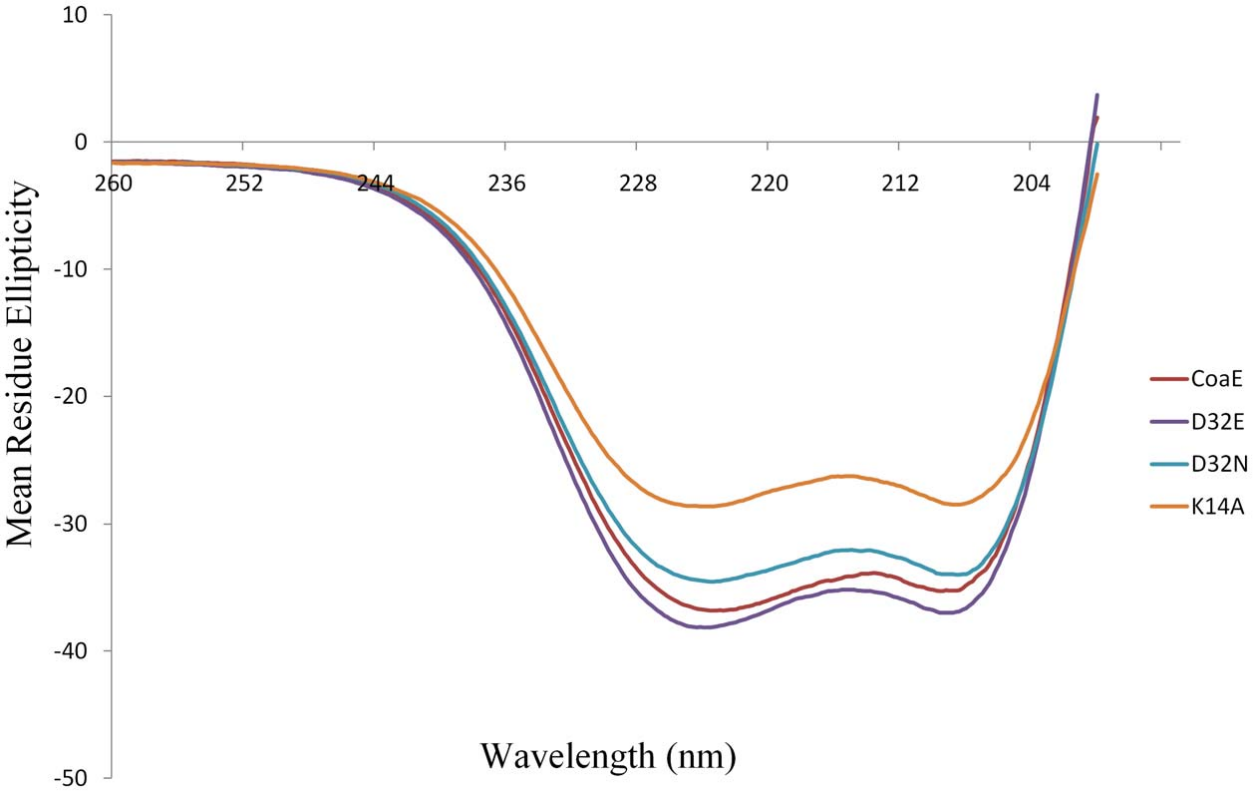
Secondary structural characterization of the mutants: Far UV CD spectra of the native CoaE and its mutants. For each mutant protein at 10 µM concentration in 10 mM phosphate buffer, pH 8.0, an average of three scans were recorded scanning from 195 nm to 260 nm.

**Figure 4:**
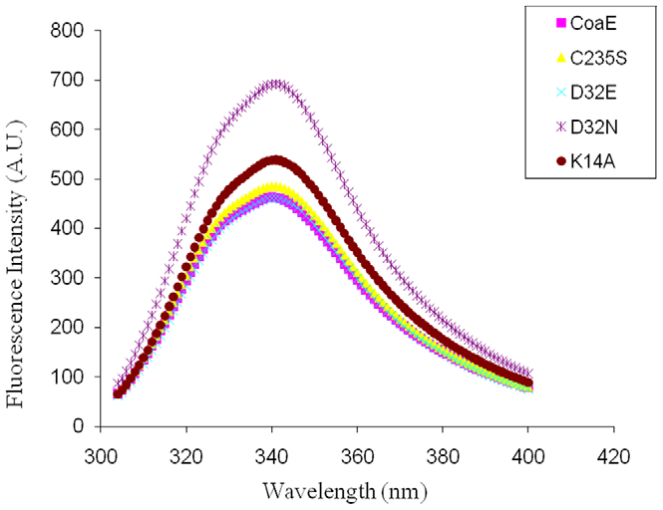
Fluorescence emission spectra for the mutants and the wild type dephosphocoenzyme A kinase from *Mycobacterium tuberculosis* excited at 295 nm and recorded at 300–400 nm.

Using the primers mentioned in Table 1, the mutants of the mycobacterial CoaE were generated either by the overlap PCR method or the full-vector amplification method as described in the Experimental Procedures section. All wild type and mutant CoaEs displayed almost identical elution profiles on SDS-PAGE demonstrating similar levels of yield, purity and migration distances. These results demonstrate that the mutants do not show any gross structural variations from the wild type enzyme. In order to rule out any changes in the secondary structure of the mutant proteins in comparison to the wild type, far UV CD studies were performed on the mutant proteins. The native enzyme is a highly structured protein with a CD spectrum typical of a protein with a high degree of α-helical secondary structure, showing minima at 209.6 nm and 223.2 nm. The α-helical content of the protein was estimated to be 37% helix, 26% β-sheet, and 38% random coil. Except the K14A mutant which showed a loss of approximately 18% of the secondary structure, all mutants showed similar values of the secondary structural components as the wild type enzyme (Figure 3). Fluorescence of endogenous tryptophan residues is sensitive to their local molecular environment, therefore the 11 tryptophan residues of mycobacterial CoaE can be used to probe conformational changes in the protein, if any. All the mutants show emission maxima at 337.5 nm as is shown by the wild type enzyme (Figure 4). Though the other mutants showed similar fluorescent intensities as the wild type, the D32E mutant showed a higher intensity, which further reflects in its kinetic properties and has been discussed in the next section.

**Table 1:** Sequences of primers used to clone the mutants of mycobacterial dephosphocoenzyme A kinase.

Arg140Lys_Fwd	5′-CGGGTGCGAAAGCTTGTCGAGCAACGCGGCAT- 3′
Arg140Lys_Rev	5′ –AAGCTTTCGCACCCGTAGCTCGACGTCGGCGTG- 3′
Arg140Ala_Fwd	5′ –GGTGCGAGCGCTGGTCGAGCAACGCGGCAT- 3′
Arg140Ala_Rev	5′- GACCAGCGCTCGCACCCGTAGCTCGACGTCGG- 3′
Asp32Glu_Fwd	5′ –GTCGACGGCGAAGTGTTGGCCCGGGAAGTG- 3′
Asp32Glu_Rev	5′ – CAACACTTCGCCGTCGACAACGATTCCGCCGC- 3′
Asp32Asn_Fwd	5′- GTCGACGGCAATGTGTTGGCCCGGGAAGTG- 3′
Asp32Asn_Rev	5′ –CAACACATTGCCGTCGACAACGATTCCGCCGC- 3′
Asp32Ala_Rev	5′ –GCGCCAGTACTGCGCCGTCGACAACGATTCCGCCGC- 3′
Asp32Ala_Fwd	5′ –GGCGCAGTACTGGCGCGTGAAGTGGTCCAG- 3′
Lys14Ala_Fwd	5′ –CGGGGCGTCGTTGCTGTCCACGACG- 3′
Lys14Ala_Rev	5′- ACGACGCCCCGGCCAATGCCGCC- 3′
Leu114Ala_Fwd	5′ –GAAGATATCCCAGCGCTGGTGGAATCCGGGATG- 3′
Leu114Ala_Rev	5′ –CCAGCGCTGGGATATCTTCGACCACAACCGCGTCC- 3′
Gly8Ala_Fwd	5- GCGGCCGGTCGGGCTACGCGTCGTA- 3′
Gly8Ala_Rev	5′-CAGTGCGATATCTTCGACCACAACCGCGTCCC- 3′

### Limited Proteolysis

Short exposure to proteases allows the latter to cleave the native enzyme at select sites on the surface of the folded protein, generating a specific pattern of the digested fragments unique to each protein-protease pair. A similar tryptic cleavage pattern for the wild type enzyme and all the mutants was indicative of retention of the overall three-dimensional geometry of the enzymes, suggesting identical structures.

Identical behavior of these mutants in CD and fluorescence measurements as well as limited proteolysis assays ruled out the possibility of global conformational changes induced upon mutagenesis as the cause for the variations in the kinetic and binding parameters.

### Kinetic Analyses

**Figure 5:**
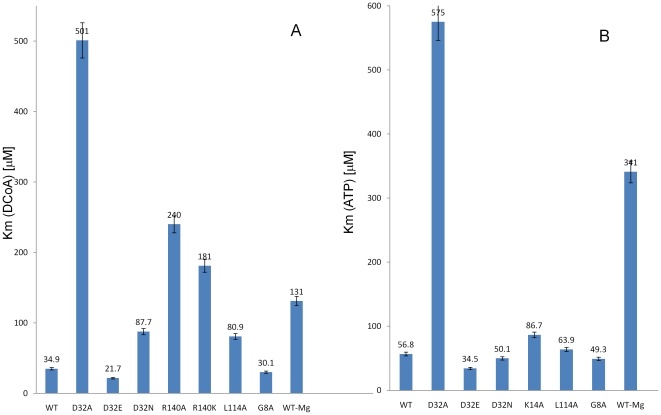
Effects of the mutagenesis on the kinetic parameters of the mycobacterial dephosphocoenzyme A kinase. The kinetic values depicted are a mean of values ±S.E. of two independent experiments performed in duplicates. A. Comparison of the Km values of the mutants for DCoA with that of the WT, B. Comparison of the Km values of the mutants for ATP with that of the WT. WT-Mg and D32N-Mg entries refer to the Km values obtained in experiments carried out in the absence of magnesium.

Kinetic analyses on all mutants were carried out by the single-coupled α-ketoglutarate dehydrogenase assay system. In order to further verify the kinetic parameters by a more direct method, ITC kinetic studies were undertaken for each mutant (13). Both the assay methods yielded similar results for the kinetic parameters (Figure 5). Further, each mutant was tested for its binding energetics with respect to the two substrates, dephosphocoenzyme A and ATP.

### Residues in the nucleotide-binding domain

Like all nucleotide-binding proteins, CoaEs possess the Walker A sequence motif, G-X-X-X-X-G-K-S/T (X =  any amino acid), which is instrumental in forming the phosphate-binding loop (P-loop) involved in binding nucleotide phosphates (14). A highly conserved feature of the ATP- and GTP-binding proteins, the P-loop is generally located at the point where a β-strand joins an α-helix forming a large anion hole with a positive electrostatic potential provided by the amino groups of the loop and the dipole of helix α1, where phosphate groups of the nucleotides bind. This motif is implicated in the cleavage of the β-γ phosphate bond. The N-terminal glycine and the C-terminal lysine of this motif are absolutely conserved across species, with variations seen in the intervening residues (15). Therefore the criticality of the P-loop glycine in the mycobacterial enzyme was tested first.

#### Gly8Ala

The glycine-rich region of the P-loop provides this structural motif with conformational flexibility. The invariable N-terminal glycine was mutated to alanine and this mutation did not change either the Km or the Kcat of the reaction considerably (Km for DCoA being 37.2 µM and that for ATP, 59.8 µM). This is understandable in light of the fact that glycine is involved in merely providing structural flexibility allowing the neighboring amino acids to form backbone hydrogen bonds with the β- and γ-phosphates of the NTP. This is further corroborated by the fact that P-loops of several proteins have an initial alanine instead of a glycine, e.g. the P-loops of elongation factors, some phosphoglycerate kinases and a thymidine kinase (16).

The invariable P-loop lysine, which hydrogen-bonds with the NTP γ-phosphate, plays a significant conformational role in stabilizing the pentavalent transition state of the γ-phosphoryl group during the phosphoryl transfer reaction. This lysine has been shown, at some instances, to tolerate a conservative substitution to arginine (17, 18). A non-conservative substitution to alanine has, however, shown conflicting results with some mutant enzymes completely losing activity while others showing no effect on the Km of the enzyme (16). Therefore we chose to substitute the lysine in the mycobacterial dephosphocoenzyme A kinase P-loop 8-(GGIGAGKS)-15 to the non-homologous alanine residue to check the essentiality of this residue and its tolerance to change.

#### Lys14Ala

**Figure 6:**
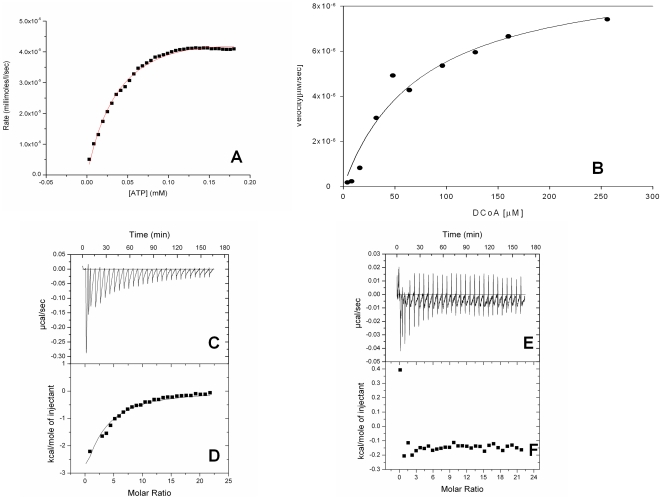
Representative profiles for mutant enzyme kinetics (by a coupled kinetic assay and ITC) and binding measurements. *A*. D32N enzyme kinetics with ATP as calculated by the ITC assay. 1.4 mL of 1 µM D32N (dialyzed 12–18 hrs in 25 mM Tris buffer, pH 7.8, 150 mM NaCl, 10 mM MgCl_2_, 20 mM KCl, 5% glycerol) was preincubated with 1 mM DCoA for 30 mins and was titrated against 1 mM ATP (298 µL) at 20°C. Raw data were collected for substrate heats of dilution in the buffer and integrated using the Microcal Origin 7.0 software. *B*, K14A enzyme kinetics with Dephosphocoenzyme A, as calculated by the coupled α-ketoglutarate dehydrogenase assay. *C* and *D*, titration of 1 mM DCoA against 100 µM K14A, *E* and *F*, titration of 1 mM ATP against 1 mM DCoA-saturated R144A showing that the R144A mutant shows poor binding to ATP. Raw data are shown as differential power signals in *C* and *E*. In *D* and *F*, the area under the curve produced on each injection was integrated (*filled squares*) and was plotted against the molar ratio of DCoA to enzyme binding sites using the Origin 7.0 software. The *solid lines* represent nonlinear best fits for a single-site binding model. Titrations were performed in 50 mM Tris/HCl, 150 mM NaCl, 5% glycerol, pH 7.8 at 20°C.

Interestingly, the substitution of the polar, basic lysine to the non-polar, hydrophobic alanine did not completely ablate enzymatic activity (Figure 6B). The substitution, though, affects the kinetic parameters of the reaction resulting in a mere 19% reduction in the Kcat of the enzyme (2.34 min^-1^ as against 2.886 min^-1^ for the WT). This 19% reduction in the Kcat correlates well with the minor loss of structure for this mutant as seen in Figure 3, which is also 18%. The K14A mutant, demonstrated a 50% increase in the Km for ATP with a Km value of 86.7 µM as against 56.8 µM for the WT (Table 2). We further investigated the effects of this mutation on the binding interactions of the mutant enzyme with the substrates. As previously reported, the native mycobacterial CoaE shows an ordered mode of substrate binding with DCoA acting as the leading substrate and ATP following it. K14A bound DCoA with almost the same affinity as the native enzyme (ΔH = -3576 calM^−1^, Ka = 1.32*10^−4^ M^−1^) (Figure 6C and D, Table 3). On the other hand, the binding of the non-hydrolyzable analog of ATP on DCoA-bound enzyme differed from that of the WT, the former reaction being mostly entropically driven with a smaller enthapic component as compared to the latter reacftion (ΔH = −1826 calM^−1^, Ka = 2.36*10^−4^ M^−1^, ΔS = 13.8 calM^−1^K^−1^ for K14A as against ΔH = −3719 calM^−1^, Ka = 1.43*10^−4^ M^−1^ΔS = 6.32 calM^−1^K^−1^ for CoaE) (Figure 6E and F).

**Table 2:** Kinetic parameters of the wild type and mutant mycobacterial dephosphocoenzyme A kinases (nr- no reaction; nd-not determined).

	DCoA			ATP		
	Km(µM)	Kcat (min^−1^)	Kcat/Km (µM^-1^ min^−1^)	Km(µM)	Kcat (min^−1^)	Kcat/Km (µM^−1^ min^−1^)
WT	34.9 (±3.2)	1.758	0.0503	56.8 (±4.8)	2.886	0.0508
K14A	75.8 (±3.8)	1.532	0.0202	86.7 (±3.9)	2.34	0.0269
G8A	37.2(±2.9)	1.543	0.0414	59.8(±2.6)	2.563	0.0428
R140A	Nr	nr	-	nr	nr	-
R140K	181(±8.1)	0.453	0.00250	nd	nd	-
D32A	501(±23.2)	0.1026	0.0000204	57.5(±2.7)	0.84	0.0146
D32E	21.7(±1.2)	1.962	0.0904	34.5(±2.9)	3.996	0.1158
D32N	87.7(±3.9)	2.004	0.0228	50.1(±3.2)	3.906	0.0779
L114A	80.9(±4.2)	3.414	0.0422	63.9(±3.9)	1.152	0.0180
WT-Mg	131(±11.2)	1.542	0.0117	341(±10.9)	0.762	0.00223
D32N-Mg	nr	nr	-	nr	nr	-

WT-Mg and D32N-Mg entries refer to the parameters calculated in the absence of magnesium.

**Table 3:** Thermodynamic parameters of the binding of the substrates, DCoA and ATP to the wild type and mutant mycobacterial dephosphocoenzyme A kinases at pH 7.8.

Titrant	*N*	*ΔH (calM^−1^)*	K_a_ (M^−1^*10^−4^)	*ΔS (calM^−1^K^−1^)*
CoaE bind DCoA	0.810	−3719((±201.5)	1.43((±0.34)	6.32(±0.34)
CoaE(DCoA) bind NHATP	0.947	−3776((±234.5)	2.06((±0.47)	6.85(±0.48)
K14A bind DCoA	0.865	−3576(±115.7)	1.32(±1.45E3)	5.73(±0.37)
K14A(DCoA) bind NHATP	1.146	−1826(±195.7)	2.36(±3.83E3)	13.8(±0.59)
R140K bind DCoA	0.527	−7723	2.17(±1.23E3)	−6.5((±0.49)
R140K(DCoA) bind NHATP	No binding	-	-	-
D32E bind DCoA	1.04	−4355(±138.7)	2.12 (±2.61E3)	4.94(±0.42)
D32A bind DCoA	0.878	−1559(±72.9)	7.99E3	−35.1(±0.98)
R140K (DCoA)bind AMPPMP	No binding	-	-	-

Bracketed ligands next to the enzymes refer to the substrate against which the enzyme was pre-saturated before the titration. Values of ΔH and ΔS are in calM^−1^ and calM^−1^K^−1^ respectively. Values are the mean of five individual experiments. K_a_ is the binding constant determined by ITC and its values are in M^−1^.

Despite the fact that the substituted residue differs from lysine both in terms of its size and structure as well as its charge, charge stabilization during phosphoryl transfer has been compensated for, in the mutant enzyme. Also, the differences in the binding energetic of the K14A mutant from the WT hint at altered interactions in the mutant enzyme. Similar results have been previously demonstrated by Driscoll et al. for a Lys-Ala substitution in the conserved GXXGXXK motif of the guinea pig estrogen sulfotransferase, which is essential for binding the activated sulfonate donor 3′-phosphoadenosine 5′-phosphosulfate (PAPS) (19). Driscoll et al. reasoned that the positive charge on a nearby arginine (Arg258) in gpEST could be positioned so as to effectively compensate for the loss of K266 upon mutation to alanine, thus leaving the Km for PAPS unaffected. From the modeled structure of the mycobacterial CoaE, Arg140 which is close to the lid domain could possibly perform this function. Therefore, we chose to mutate this residue next.

Therefore, though the P-loop has a consensus sequence conserved across kinases from several species and plays a significant role in nucleotide binding, the residues constituting the P-loop of each kinase demonstrate varying levels of resistance to perturbation. As elaborated by Saraste et al., disturbance at the P-loop has a varying range of effects at each position across species, these effects being influenced by the conformation and flexibility of the rest of the protein despite the three-dimensional structure of the P-loop itself being highly conserved (16).

### Residues in the lid domain

The CoA domain and the lid domain are mobile as can be seen from the high atomic temperature factors for the residues constituting these domains in the crystallographic structures of dephosphocoenzyme A kinases. Region Thr141-Ile155 of the *E. coli* enzyme has a higher temperature factor as it forms the Lid domain and participates in catalysis. This includes the Arg140 residue that forms salt bridges with the ATP phosphates and van der Waals bonds with the ribose.

#### Arg140Ala

The arginine residue was substituted with the uncharged alanine to check for the role of this residue in charge stabilization. Strikingly, this mutation completely abolishes kinetic activity. Since the uncharged alanine cannot compensate for the charge stabilization during phosphoryl transfer, these results are consistent with a role for the positive charge of Arg140 in ATP binding and stabilization of the phosphoryl intermediate during catalysis. In order to further study the role of this arginine residue, we mutated it to the homologous lysine, which though differs in structure, can possibly provide the charge stabilization afforded by the arginine residue at this position.

#### Arg140Lys

Strikingly, the conservative homologous polar basic residue, lysine also could not substitute for the Arg at position 140 resulting in a dramatic loss of catalytic activity, with a Kcat of 0.453 min^−1^, barely 15% of the WT and a Km for the leading substrate, DCoA, 518-times that of the WT. A striking decrease in the Kcat/Km ratio for the mutant (0.00250 µM^−1^ min^−1^, Table 2) points out its critical role in enzyme catalysis. In order to further elucidate the interaction of this mutant with the enzyme's substrates, we carried out ITC binding studies. Interestingly, the R140K mutant bound the leading substrate, DCoA reasonably well with ΔH and Ka values similar to those of the WT (Table 2), but this mutant showed very poor binding to ATP, thereby explaining the loss of activity for this mutant.

The mycobacterial CoaE Arg140 is structurally equivalent to the Arg124 of the *E. coli* gluconate kinase (GntK), Arg140 from the *H. influenzae* dephosphocoenzyme A kinase and Arg127 in adenylate kinase from *Bacillus stearothermophilus* (5, 10, 20). Arg140 from *Haemophilus* has been shown to be important for the formation of the ATP binding pocket by linking to the P-loop carbonyl groups and forms the ATP adenine binding site. The crystal structure of the GntK-ATP binary complex clearly shows that the side-chain of Arg124 is shifted in the complex with the nucleotide and is positioned into interacting distance of ATP, possibly performing a corresponding protective role during catalysis. In a similar analogy, in the mycobacterial CoaE, conformational changes induced upon DCoA binding (which as has been established previously, binds first), could in all probability, bring Arg140 closer to the ATP phosphate groups. In this conformation, the arginine residue now performs a dual function like its counterparts in the *B. stearothermophilus* and *E. coli* enzymes, of both stabilizing the transition state as well as protecting the phosphoryl groups from solvent exposure. Unfortunately, both the substitutions at this residue, could not possibly shield the γ-phosphate of ATP from a nucleophilic attack from the omnipresent water. Substitution to alanine resulted in the loss of charge and the lysine replacement, though similar in polarity and charge, was conformationally constrained and failed to compensate for the bulkier residue at this position preventing it from contacting the phosphoryl intermediate during the reaction. Therefore these results clearly show that the Arg140 residue is essential at its position and it not only participates in a charge-charge interaction with the ATP phosphoryl groups, but the guanidinium group of arginine is also required for its function, such as via a specific hydrogen bond network or stacking interaction with the adenine base, thereby contributing to base localization and recognition. Therefore, Arg140 from the lid domain participates in substrate binding and stabilization of the reaction intermediate and plays a key role in the phosphotransfer apart from Lys 14 from the P-loop.

### Residues in the CoA domain

Crystal structures of homologous enzymes like adenylate kinase, creatine kinase, gluconate kinase etc have revealed carboxylate groups within 2.8 Å of the proposed transphosphorylation site. From our docking studies, Asp32 emerged as a possible catalytic residue by virtue of it being oriented such that its carboxyl group is within hydrogen bonding distance from the 3′-OH of the substrate and therefore acts as the general base that activates a nucleophile to initiate attack on the ATP γ-phosphate (Figure 2). Also, in the protein-ligand interaction analysis undertaken previously, this residue shows a strong interaction with the leading substrate, DCoA, making 15 contacts with the latter (6). Therefore, we chose to study the role of Asp32 in promoting nucleophilic attack on the γ-phosphate of ATP by substituting this residue with three other amino acids; in order to probe its potential role in catalysis.

#### Asp32Ala

A non-conservative substitution of the chief catalytic residue at position 32 led to drastic effects on the kinetic parameters with a 15-fold increase in the Km for DCoA (501 µM) (Table 2). The D32A showed a catalytic efficiency of only 5% of the native enzyme, indicating a crucial role played by this residue in catalysis. Interestingly, the Km of the D32A mutant for ATP remained similar to that of the WT, though the Kcat dropped drastically to about 28% of the WT. No considerable change in the Km, a decreased Kcat and a drastically reduced Kcat/Km ratio of a mere 0.0000204 µM^−1^ min^−1^ imply a decreased efficiency and differences in enzymatic catalysis by this mutant, suggesting the possibility of a decreased stability of the transition state. Assuming the role of the catalytic aspartyl in facilitating the nucleophilic attack, its absence would result in product-release being the rate-limiting step of the kinetic reaction. Therefore the substitution of an uncharged amino acid at this crucial position would compromise the release of a negatively-charged product. The Kcat of 0.84 min^−1^ can therefore be assumed to have arisen as a result of the small size of the alanine residue allowing a water molecule to occupy the space in the active site and thereby partially fulfilling the role of a nucleophile originally performed by the native enzyme aspartate carboxyl. The circular dichroism spectra of the D32A variant varied by only 3.6% at 220 nm confirming that an alanine replacement of the active site Asp does not affect the conformation significantly and therefore the mutation results only in the loss of an essential functional group acting as a base in the phosphate-transfer reaction.

#### Asp32Asn

In order to minimize effects due to a change in the size and structure at a key catalytic position, we chose to substitute the Asp32 residue with the most conservative replacement in terms of molecular volume and polarity, an asparagine. The D32N mutant shows a Km of 87.7 µM for DCoA, almost 2.54-times the WT. The Kcat for the reaction was 2.004 min^-1^. Interestingly, substitution to the corresponding amide, the uncharged asparagine did not affect the Km of the mutant for ATP with the Km being 50.1 µM (Figure 6A). The Kcat for this reaction was 3.906 min^−1^. This implies that Asp32 is highly specific and essential for enzymatic activity.

#### Asp32Glu

Striking results of mutagenesis at this residue were obtained upon a functionally conservative substitution to glutamic acid, which resulted in a 62.17% increase in the affinity of the mutant enzyme for the leading substrate, DCoA and a 60.58% increase in the affinity for ATP, the Km values being 21.7 µM and 34.5 µM respectively. The D32E mutant, which has the same functional group as the native residue at this position has a very minimal effect on the Kcat of the mutant enzyme. As observed during our previous DCoA-docking analyses as well those by Obmolova et al., the DCoA molecule is oriented such that its ribose projects the 3′OH within hydrogen bonding distance to the catalytic aspartate, the distance between the hydroxyl and ATP γ-phosphate being 7 Å (5, 6). As seen in all NMP kinases, major conformational rearrangements upon lid domain closure and catalysis bridge this distance, allowing catalysis. Therefore, in a similar analogy to the *H. influenzae* CoaE, an increase in the Km values of the D32E mutant is possibly observed due to the lengthening of the carbon chain of the catalytic carboxyl by a methylene in the glutamate which reduces the observed 7 Å distance between the 3′-OH and the catalytic residue, aiding faster transphosphorylation. This is further evidenced from the increase in fluorescence intensity for this mutant in comparison to the WT (Figure 4). This increase in fluorescence is perhaps due to a compaction of the hydrophobic core which leads to an increase in the affinity for its substrates. These findings are not surprising because several kinases like the mammalian muscle creatinine kinase and arginine kinase have glutamates close to the transphosphorylation site whose carboxyl groups carry out the nucleophilic attack on the γ-phosphate of ATP (21, 22). Therefore, enzymes that are relatively more evolved in the evolutionary tree probably utilize glutamic acid as the chief catalytic residue. This is substantiated by the observed decrease in the Km of the D32E mutant enzyme.

In our previous homology modeling and docking analyses, DCoA docked on the modeled CoaE fits snugly in the deep cleft between the core and the CoA domain. The adenine base of DCoA bound deep in the cleft in a hydrophobic environment provided by Ala 35, Val91, Ile112, Leu114, Leu115, and Ala158. We chose to study the role of the Leu114 residue in DCoA binding as it is the most conserved residue amongst the residues that line the hydrophobic cleft into which the acceptor substrate binds. Its significance is further corroborated by the fact that this residue forms 16 contacts with DCoA, as seen previously in our LPC analysis (6). Furthermore, this leucine is the only residue that differs in the human and the mycobacterial dephosphocoenzyme A kinase in this hydrophobic cleft, the rest of the residues being conserved. Therefore we chose to study this residue as its characteristics can shed light upon the differences between the host and the pathogen enzymes.

#### Leu114Ala

A non-conservative substitution of this highly conserved residue resulted in a decrease in the affinity of the enzyme for the acceptor substrate, with a Km value of 80.9 µM as against 34.9 µM for the WT. Interestingly the mutant enzyme compensates for the drop in affinity by increasing the enzymatic turnover with a Kcat value of 3.414 min^−1^ as against 1.758 min^−1^ for the WT.

### The role of Magnesium ions

Almost all members of the P-loop containing NTP hydrolase superfamily bind ATP as the ATP-Mg^2+^ adduct. This divalent metal ion coordinates with the hydroxyl of the P-loop serine/threonine residue, the β- and γ-phosphates of ATP and three water molecules, which hydrogen bond with the carboxylates of two chief catalytic aspartates/glutamates (5). Mg^2+^ participates in catalysis by stabilizing the negative charges on the phosphates and creating the optimal geometry for inline phosphotransfer by orienting the ATP γ-phosphate. All the sequence and structural homologs of the mycobacterial dephosphocoenzyme A kinase, such as the *Haemophilus* CoaE, the adenosine-5′-phosphosulphate kinase and shikimate kinase, have two aspartic acid residues in close proximity to each other, which build the coordination sphere of the magnesium ion (5). A similar role in the mycobacterial enzyme could be performed by the Asp 30 and Asp32 residues of the DXD motif (6). Therefore, in order to study the role of the Asp32 carboxylate in coordinating the divalent ion for phosphoryl transfer, we carried out the enzymatic assay of both the wild type enzyme and the mutants in the absence of magnesium. Kinetic assays for the WT enzyme in the absence of Mg^2+^ showed an increment of the Km for ATP with a concomitant decrease in the catalytic efficiency. On the other hand, the absence of magnesium completely ablates activity for the D32N mutant. When magnesium was externally supplemented for the reaction, the D32N mutant regains its original activity, as mentioned above. Therefore the carboxylate at position 32 of the mycobacterial enzyme is critical for coordinating Mg^2+^ for the phosphoryl transfer reaction and like other P-loop containing enzymes, the mycobacterial CoaE also requires magnesium for optimal activity.

### Conclusions

The lack of information on the three-dimensional structure of the mycobacterial dephosphocoenzyme A kinase has precluded a clear definition of the functional residues involved in ligand binding and catalysis. Information is lacking on the exact mechanistic details of the tubercular enzyme which belongs to the NTP hydrolase superfamily. Notwithstanding their primary sequence differences, all enzymes belonging to this superfamily possess the three-domain fold and behave similarly in terms of domain movements upon substrate binding and catalysis. Therefore based on the sequence and structural homology of the modeled mycobacterial CoaE, we chose residues from all the three domains of the mycobacterial CoaE to identify their functional contributions. We have identified the residues involved in binding ATP (Lys14), binding the leading substrate (Leu114), stabilizing the phosphotransfer reaction (Arg140), coordinating with the divalent cation Mg^2+^ (Asp32) and promoting the nucleophilic attack on the γ-phosphate of ATP (Asp32), biochemically demonstrating for the first time the importance of these residues in a dephosphocoenzyme A kinase. It should be noted that even though X-ray crystallographic data often provides conclusive evidence in favor of a given mechanism, the latter remains a proposal till proven by detailed biochemical (mutagenic) studies. Therefore these studies conclusively assign functional roles to the residues identified in the three crystal structures of CoaEs currently available. Further, we show that the mycobacterial enzyme requires the divalent cation magnesium for its catalytic activity. We initially established that the changes in the kinetic and binding parameters were not in consequence of the conformational changes induced in the mutant proteins by ensuring similarity of the mutant proteins to the WT by CD and fluorescence measurements as well as by the susceptibility to trypsin cleavage, all of which indicate a common overall three-dimensional structure. Our results demonstrate that the residue Arg140 is strictly conserved while residues at the positions, Gly8, Asp32, Lys14, Leu114 can tolerate substitutions to a certain extent. Further, by virtue of the definition of a catalytic residue, as described by Bartlett et.al, Asp32 and Arg140 can be classified as catalytic residues (23). Therefore, this study presents a thorough molecular dissection of the roles played by crucial amino acids of the protein. Though, a full understanding of the complete mechanistic picture awaits a detailed three dimensional analysis of the wild type and the mutants presented in this paper, the results herein can serve as a good starting point for targeted drug development approaches. A similar detailed study of the human enzyme would further highlight the differences between the two enzymes and take us closer towards the universal goal of efficient drug design against this dreaded pathogen.

## Materials and Methods

### Materials

Unless otherwise stated, chemicals and reagents were purchased from Sigma-Aldrich Chemicals. Restriction enzymes were purchased from New England Biolabs.

### Bacterial Strains and Vectors

*coaE* wild-type construct was created as previously described (6). *coaE* mutant plasmid DNA was transformed into DH5α cells for mutant construction, propagation, and sequencing. The vector constructs carrying the desired mutation were then transformed into BL21 (DE3) competent cells.

### Site-Directed Mutagenesis

Employing the primers mentioned in Table 1, amino acid substitutions were generated by either of the two methods: For mutagenesis by the mutated primer method, the target gene, CoaE, cloned into the pET28a+ vector was used as the template. Custom synthesized oligonucleotide primers with the desired mismatches were used to incorporate the required silent mutation, preferably incorporating alongside, a restriction enzyme recognition site for future clone confirmation. After full vector amplification, the PCR product was treated with DpnI at 37°C for 2 hours. Post enzyme inactivation at 80°C, the mixture was cleaned up and transformed into *E. coli* DH5α. For mutagenesis by overlap extension, in separate PCR reactions, two fragments of the target sequence were amplified by using, for each reaction, one universal (the original CoaE primer) and one mutagenic primer. The two intermediate products with terminal complementarity, were gel purified and used to generate a new template DNA by duplexing in a second reaction. During the overlap extension phase, the fused product was amplified using the two original CoaE primers. Conditions were standardized for all the 4 separate PCR reactions. The resulting full length mutant gene fragments were then ligated with the pET28a+ vector and transformed into *E. coli* DH5α. After screening for mutants, the mutant DNA was sequenced and transformed into BL21 cells.

### Expression and purification of mutant proteins

Plasmids containing the *coaE* gene were transformed into BL21(DE3) and spread onto LB agar plates containing kanamycin (100 µg/mL). Single colonies were used to inoculate a 100 mL primary culture which was used further to inoculate a 6 L secondary culture in LB medium containing the appropriate antibiotics. Cells were grown in an incubator/shaker at 37°C to an absorbance reading (A_600_) of 0.7–0.8 at which point IPTG (0.4 mM) was added and the flasks were cooled to 18°C for overnight growth (∼12 hr). Cells were harvested by centrifugation (15 min at 6000*g*), the cell pellet was washed with 25 mM Tris, 150 mM NaCl, 5% glycerol buffer, pH 7.8, collected and stored at −20°C.

### Western Blot Analysis

The hexahistidine-tagged mutant enzymes were run on a 10% SDS-PAGE, transferred onto nitrocellulose membranes and confirmed by detection with Anti-His antibodies.

### Circular dichroism studies

All the CD experiments were done in a JASCO-J715 polarimeter (JASCO, Tokyo, Japan) in a 0.1-cm pathlength cell, with a slit width of 1 nm, response time of 4 s and a scan speed of 50 nm/s. Each data point was an average of three accumulations. Each mutant enzyme was dialyzed overnight in phosphate buffer (140 mM Nacl, 2.7 mM KCl, 1.8 mM KH_2_PO_4_, and 10 mM Na_2_HPO_4_) containing 5% glycerol, pH 7.4. 5 µM of each mutant was then scanned from 260 nm–195 nm to study its secondary structural elements.

### Fluorescence measurements

Steady-state fluorescence measurements were carried out on a Jobin Yvon Horiba fluorometer (Jobin Yvon (Spex division), Cedex, France) using a protein concentration of 7.5 µM in 10 mM phosphate buffer, pH 8.0. The excitation wavelength was kept fixed at 295 nm and the emission spectra were recorded from 305 to 450 nm. The monochromator slit width was fixed at 5 nm for both excitation and emission.

### Susceptibility to Proteolytic Digestion

Both the wild type dephosphocoenzyme A kinase and all the mutants were checked for their susceptibility to tryptic digestion by checking their fragmentation patterns upon exposure to trypsin under limiting conditions at a protease to protein ratio of 1∶50 (w/w). Aliquots were withdrawn after 10 and 30 mins and were run on a 10% SDS-PAGE for separation of the proteolysis products.

### Enzyme Assays

All enzyme assays were carried out at 25°C. Units of activity correspond to 1 µmol of product formed/min, and any blank rates without DCoA, taken as controls, were subtracted. CoaE activity was assayed using two different coupled assays; the established double coupled pyruvate kinase/lactate dehydrogenase (PK/LDH) (24) and a single-coupled α-ketoglutarate dehydrogenase (KDH) assay. The latter assays for CoA formation by monitoring an increase in absorbance at 340 nm due to NADP+ reduction. Each reaction mixture for the α-KDH assay contained ATP (10 mM), DCoA (0.5 mM), MgCl_2_ (10 mM), KCl (20 mM), α-ketoglutarate dehydrogenase (2U), α?ketoglutarate (2 mM), NADP (0.3 mM), thymine pyrophosphate (0.3 mM) in 50 mM Tris, pH 8.0. Reactions were carried out by using different concentrations of DCoA (Set 1), ATP (Set 2) and CoaE (Set3) by monitoring the change in absorbance at 340 nm (A_340_) for 5 mins each on a Jasco spectrophotometer. Kinetic parameters for different substrates were determined by global non-linear least squares fitting of initial rate data. For checking the role of magnesium in enzymatic activity, both CoaE and the mutant enzymes, were dialyzed in a buffer lacking Mg^2+^ and the results of the kinetic analyses with these enzymes were compared to those from enzymes dialyzed in buffers containing Mg^2+^.

### ITC experiments

ITC experiments were performed using a VP-ITC titration microcalorimeter (Microcal Inc., U.S.A.). The reference cell was filled with water and the calorimeter was calibrated using standard electrical pulses as recommended by the manufacturer. For all ITC experiments, the CoaE protein was dialyzed overnight (12–18 hr) in 10 mM Tris buffer, pH 7.8, 150 mM NaCl, 10 mM MgCl_2_, 20 mM KCl and 5% glycerol. Substrate solutions were prepared in the final dialysis buffer. Solutions of the protein were filled in the sample cell (1.4 mL) and titrated with ATP-γS (a non-hydrolysable analog of ATP) or DCoA solutions introduced into it from the syringe (298 µL). Except the substrate against which the parameters were being determined, the composition of the solutions in the syringe and cell were kept constant. ITC experiments were routinely performed at 20°C. Raw data were collected for substrate heats of dilution in the buffer and integrated using the Microcal Origin software version 7.0 supplied with the instrument. In control experiments, the substrates were titrated against buffer.

### Determination of the Kinetic Parameters of ATP hydrolysis by ITC

To evaluate the kinetic parameters of ATP hydrolysis, 1 µM of dialyzed protein was incubated with 1 mM of DCoA for 45 mins on an end-on rocker at 4°C for complete saturation. A single injection of 1 mM ATP (20 µL), prepared in the final dialysis buffer, was monitored for 3000 seconds. Heat change during the course of reaction was used to determine the ΔH for the reaction to evaluate the k_cat_ value for the enzyme. To determine the kinetic constants, the protocol of ligand-multiple injections was adopted, which also helped ascertain the maximum velocity for the enzymatic reaction (13). The reaction was started by injecting 20 µL of the 1 mM ATP solution into the sample cell loaded with DCoA-saturated CoaE. A gap of 120 seconds was kept between subsequent injections. Kinetic parameters with respect to DCoA were determined in a similar fashion as those for ATP.

### ITC studies for Substrate binding to the mutant enzymes

Binding studies were carried out with DCoA, CoA, ATP-γS and ADP. The sample cell was filled with 200 µM CoaE. For each binding experiment with one substrate, 1 mM solution of the ligand was introduced from the syringe into the cell containing the enzyme saturated with the other substrate. For this purpose the enzyme was incubated with the substrate to be saturated with, on an end-on rocker for 45 mins, at 4°C. Titrations were carried out by the stepwise addition of small volumes (6 µL) of the ligand solution from the constantly stirring syringe (300 rpm) into the CoaE containing sample cell with a time interval of 180 seconds between successive injections. Change in the enthalpy, K_a_ and N values for the titration were determined by non-linear least squares fit of the data using Origin Tm 7.0 software. The data were fitted to a single-site binding model by a non-linear regression analysis to yield binding constants (K_a_), enthalpies of binding (ΔH) and the stoichiometry of binding (N).
